# Acupuncture treatment for optic atrophy: A protocol for systematic review

**DOI:** 10.1097/MD.0000000000016879

**Published:** 2019-08-16

**Authors:** Ping-ping Zhou, Peng Sun, Hong-wei Liu, Yan Meng

**Affiliations:** Department of Ophthalmology, First Affiliated Hospital of Jiamusi University, Jiamusi, China.

**Keywords:** acupuncture, effectiveness, optic atrophy, safety

## Abstract

**Background:**

Optic atrophy (OPA) is a very tricky disorder. Presently, no effective management is available for this condition. Previous studies have reported that acupuncture may be effective for the treatment of OPA. However, its effectiveness is still inconclusive. Thus, this study will aim to assess the effectiveness and safety of acupuncture for OPA.

**Methods:**

A comprehensive literature search for relevant studies will be performed from the databases of PUMBED, EMBASE, CINAHI, Cumulative Index to Nursing and Allied Health Literature, Allied and Complementary Medicine Database, Cochrane Library, Chinese Biomedical Literature Database, China National Knowledge Infrastructure, and other literature sources from inception up to the present. No language limitations will be applied to all literature searches. We will consider all randomized controlled trials (RCTs) and case-controlled trials (CCTs) for assessing the effectiveness and safety of acupuncture for OPA. The primary outcomes include the rates of vision improvement and visual field improvement. The secondary outcomes consist of the increased visual field average sensitivity, pattern visual evoked potential (PVEP) amplitude, and shortened PVEP latency, as well as any expected and unexpected adverse reactions. Risk of bias assessment will be performed by Cochrane risk of bias for RCTs and Newcastle-Ottawa Scale for CCTs.

**Results:**

In this study, we will outline details of the aims and methods on the effectiveness and safety of acupuncture for the treatment of OPA.

**Conclusion:**

The results of this study will summarize the most current evidence of acupuncture for the treatment of patients with OPA.

**Dissemination and ethics:**

The results of this study are expected to be published on peer-reviewed journals. This is a literature-based study; therefore, no ethical approval is necessary.

**Systematic review registration:**

PROSPERO CRD42019135785

## 1 Introduction

Optic atrophy (OPA) is a very tricky disorder, which involves the death of the retinal ganglion cell axons caused by various eye diseases, and results in optic nerve lesions.^[1,2,3]^ It often manifests as the degeneration and disappearance of optic nerve fibers, conduction dysfunction, visual field changes, vision decrease and loss.^[4,5,6]^ In China, previous study has reported that OPA ranks as the second factor in children's visual impairment, which seriously threatens quality of life in patients with this disorder.^[7]^ Unfortunately, there are still no effective managements for treating this condition because of the complicated causes, and its poor prognosis.

Acupuncture has been utilized in clinical practice in China for thousands of years.^[8,9,10,11]^ It has been used to treat a variety of conditions and has achieved a promising effectiveness.^[12,13,14,15,16,17,18,19,20]^ Several previous studies have reported that acupuncture may also benefit for patients with OPA.^[21,22,23,24,25,26]^ However, its effectiveness for this disorder is still inconclusive. Thus, this study will comprehensively and systematically assess the effectiveness and safety of acupuncture for patients with OPA.

## 2 Methods and analysis

### 2.1 Study registration

This study has been registered on PROSPERO (CRD42019135785), and we have reported it in accordance with the guidelines of the Preferred Reporting Items for Systematic Reviews and Meta-analysis Protocol.

### 2.2 Eligibility criteria for study selection

#### 2.2.1 Types of studies

Any randomized controlled trials (RCTs) and case-controlled trials (CCTs) of acupuncture for the treatment OPA will be considered for inclusion. However, nonclinical trials, and noncontrol trials will be excluded.

#### 2.2.2 Types of patients

We will include any diagnosed criteria of OPA regardless their race, sex, and age.

#### 2.2.3 Types of interventions

We will include studies that have implemented acupuncture monotherapy as an experimental treatment.

Control therapy can be any treatments, except any forms of acupuncture therapies.

#### 2.2.4 Types of outcomes

Eligible studies will be included if more than one of the following outcomes have reported.

##### 2.2.4.1 Primary outcome

1. Rate of vision improvement;
2. Rate of visual field improvement.

##### 2.2.4.2 Secondary outcome

1. Increased visual field average sensitivity;
2. Pattern visual evoked potential (PVEP) amplitude;
3. Shortened PVEP latency;
4. Any expected and unexpected adverse reactions.

### 2.3 Search strategy for study identification

#### 2.3.1 Electronic databases searches

This study will include a comprehensive literature search from PUMBED, EMBASE, CINAHI, Cumulative Index to Nursing and Allied Health Literature, Allied and Complementary Medicine Database, Cochrane Library, Chinese Biomedical Literature Database, and China National Knowledge Infrastructure, and other literature sources from their inceptions to the present regardless any language restrictions. The search strategy for PubMed is detailed in Table 1. Identical search strategies will also be used for any other electronic databases.

**Table 1:**
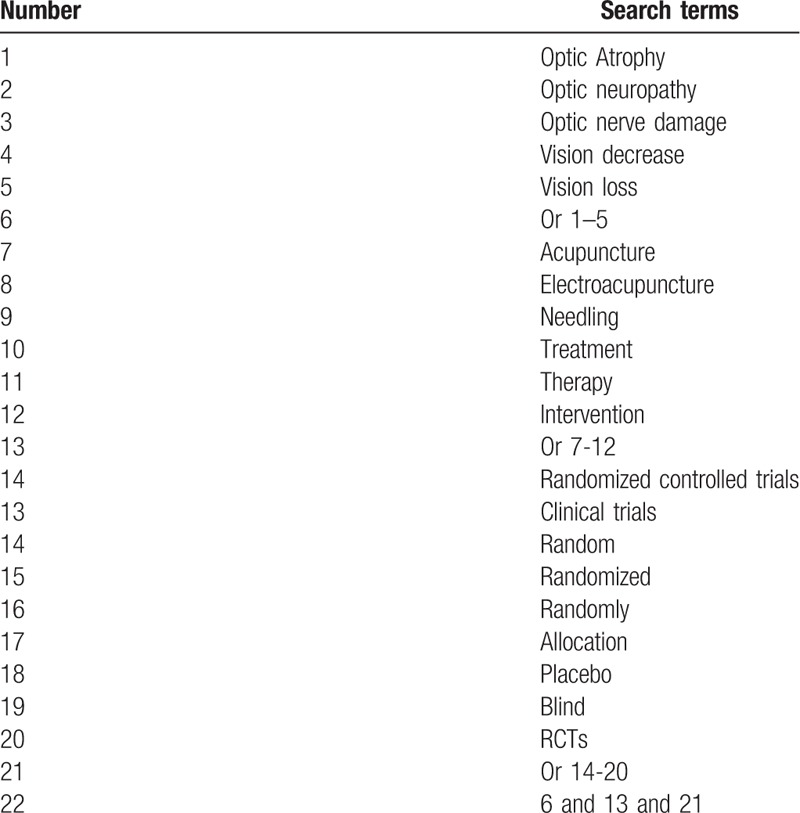
Search strategy for PubMed.

#### 2.3.2 Other literature sources

We will also retrieve any other gray literature sources, such as conference proceedings, dissertations, and reference lists of eligible studies.

### 2.4 Study selection

**Figure 1:**
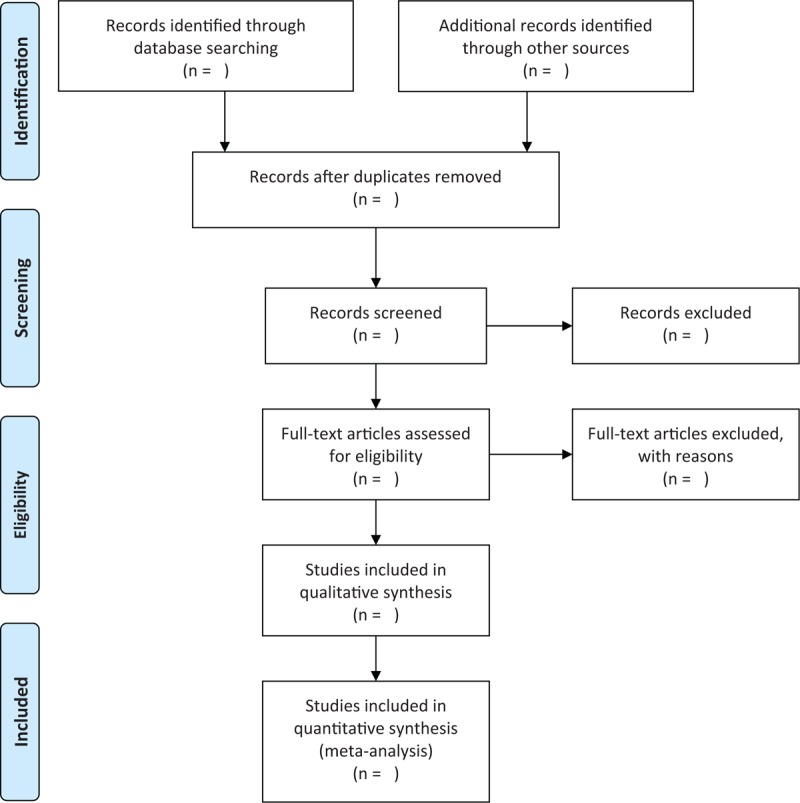
Flow diagram of study selection process.

Two independent authors will check the titles and summaries for each retrieved record according to the eligibility criteria. After that, all irrelevant records will be excluded. Then, the remaining records will be read by full-texts and will be judged if they meet the finally eligibility criteria. Any disagreements regarding the study selection between 2 authors will be solved by a third author through discussion. The flowchart of all study selection procedure is shown in Figure 1.

### 2.5 Data extraction and management

Two trained authors will independently extract the required information from all eligible studies according to the pre-designed data extraction form. A third trained author will help to check and to solve any disagreements occurred between 2 authors. Extracted information consists of publication details, general characteristics of eligible studies (such as name, title, publication year, and so on), patient details (such as sex, age, and so on), details of treatments, as well as the outcome measurements.

### 2.6 Missing data dealing with

Where applicable, any missing data will be inquired by contacting primary authors by using email. When it is not possible, only available data will be analyzed. Meanwhile, we will discuss its possible effects on the combined outcome results.

### 2.7 Risk of bias assessment

In this study, we will apply different assessment tools to evaluate different types of studies. We will use Cochrane risk of bias tool to assess the risk of bias for all RCTs, and the risk of bias for all CCTs will be evaluated by using newcastle–ottawa scale. Two trained authors will independently assess the risk of bias for each eligible study. If there are disagreements between 2 authors, a third trained author will be asked for help to solve those disagreements by discussion.

### 2.8 Rating quality of evidence

In this study, the overall strength of the evidence will also be assessed by Grading of Recommendations Assessment, Development, and Evaluation tool.^[27]^ We will summarize the results in the tables of Summary of Findings.

### 2.9 Statistical analysis

RevMan 5.3 software will be utilized for statistical analysis in this study.

#### 2.9.1 Treatment effects measurements

Continuous data will be expressed as mean difference or standardized mean difference with 95% confidence intervals.

Dichotomous data will be employed by using as risk ratio with 95% confidence intervals.

#### 2.9.2 Assessment of heterogeneity

We will assess heterogeneity by using *I* ^2^ test. If *I* ^2^ ≤50%, minor heterogeneity will be regarded. Otherwise, if *I* ^2^ > 50%, substantial heterogeneity will be observed in this study. Then, subgroup analysis will be conducted to identify any possible reasons that can result in substantial heterogeneity.

#### 2.9.3 Data synthesis

If minor heterogeneity is found (*I* ^2^ ≤50%), a fixed-effects model will be employed for data pooling, and meta-analysis will be carried out. If significant heterogeneity is found (*I* ^2^ > 50%), a random-effects model will be employed for data pooling. At the same time, subgroup analysis will also be conducted. If there is still substantial heterogeneity after subgroup analysis, data will not be pooled, and meta-analysis will not be carried out. Instead, we will report narrative summary for outcome results.

#### 2.9.4 Subgroup analysis

Subgroup analysis will be applied in accordance with different characteristics, treatment types, and outcome tools.

#### 2.9.5 Sensitivity analysis

Sensitivity analysis will be conducted to identify the robustness and stability of pooled outcome results by removing low quality of studies.

#### 2.9.6 Reporting bias analysis

If >10 qualified studies are included, Funnel plot and Egger regression analysis will be carried out to check the publication bias.

## 3 Discussion

OPA is a very common and tricky disorder, and can result in vision decrease or even loss, which significantly affect their quality of life. Several previous studies have reported that acupuncture can treat OPA effectively and safety.^[12,13,14,15,16,17,18,19,20]^ However, its effectiveness for treating this disorder is still inconclusive. Therefore, this study will systematically assess the effectiveness and safety of acupuncture for the treatment of patients with OPA. The findings of this study will summarize most recent evidence of acupuncture for treating OPA.

## Author contributions

**Conceptualization:** Ping-ping Zhou, Peng Sun, Hong-wei Liu, Yan Meng.

**Data curation:** Ping-ping Zhou, Peng Sun, Hong-wei Liu, Yan Meng.

**Formal analysis:** Ping-ping Zhou, Peng Sun, Hong-wei Liu.

**Funding acquisition:** Hong-wei Liu.

**Investigation:** Hong-wei Liu.

**Methodology:** Ping-ping Zhou, Yan Meng.

**Project administration:** Hong-wei Liu.

**Resources:** Ping-ping Zhou, Peng Sun, Yan Meng.

**Software:** Ping-ping Zhou, Peng Sun, Yan Meng.

**Supervision:** Hong-wei Liu.

**Validation:** Ping-ping Zhou, Peng Sun, Hong-wei Liu, Yan Meng.

**Visualization:** Ping-ping Zhou, Peng Sun, Hong-wei Liu.

**Writing – original draft:** Ping-ping Zhou, Peng Sun, Hong-wei Liu, Yan Meng.

**Writing – review & editing:** Ping-ping Zhou, Peng Sun, Hong-wei Liu, Yan Meng.

## References

[1] Lenaers G Hamel C Delettre C Dominant optic atrophy. Orphanet J Rare Dis 2012;7:46. 22776096 10.1186/1750-1172-7-46 PMC3526509

[2] Ishii A Ohkoshi N Optic atrophy/dystonia. Ryoikibetsu Shokogun Shirizu 2001;203–8. 11596371

[3] Wolff B Azar G Vasseur V Microcystic changes in the retinal internal nuclear layer associated with optic atrophy: a prospective study. J Ophthalmol 2014;2014:395189. 24701345 10.1155/2014/395189 PMC3950921

[4] Rönnbäck C Grønskov K Larsen M Retinal vessel diameters decrease with macular ganglion cell layer thickness in autosomal dominant optic atrophy and in healthy subjects. Acta Ophthalmol 2014;92:670–4. 24612963 10.1111/aos.12378

[5] Chamney S Jothi VG McLoone E Biotinidase deficiency, bilateral optic atrophy, and a visual field defect. Neuroophthalmology 2013;37:251–3. 28167995 10.3109/01658107.2013.824004 PMC5291002

[6] Zhang XJ Improve the understanding and diagnostic skill of optic atrophy. Ophthalmol China 2011;20:364–6.

[7] Wei QP Improve the understanding of optic atrophy. Journal of Traditional Chinese Ophthalmology 2011;21:1–3.

[8] Smith CA Armour M Zhu X Acupuncture for dysmenorrhoea. Cochrane Database Syst Rev 2016;4:CD007854. 27087494 10.1002/14651858.CD007854.pub3 PMC8406933

[9] Yin C Buchheit TE Park JJ Acupuncture for chronic pain: an update and critical overview. Curr Opin Anaesthesiol 2017;30:583–92. 28719458 10.1097/ACO.0000000000000501

[10] Zeng L Tao Y Hou W Electro-acupuncture improves psychiatric symptoms, anxiety and depression in methamphetamine addicts during abstinence: a randomized controlled trial. Medicine (Baltimore) 2018;97:e11905. 30142795 10.1097/MD.0000000000011905 PMC6112927

[11] He Y Liu Y May BH Effectiveness of acupuncture for cancer pain: protocol for an umbrella review and meta-analyses of controlled trials. BMJ Open 2017;7:e018494. 10.1136/bmjopen-2017-018494 PMC5778333 29229658

[12] Armour M Ee CC Hao J Acupuncture and acupressure for premenstrual syndrome. Cochrane Database Syst Rev 2018;8:CD005290. 30105749 10.1002/14651858.CD005290.pub2 PMC6513602

[13] Wang HQ Bao CL Jiao ZH Efficacy and safety of penetration acupuncture on head for acute intracerebral hemorrhage: A randomized controlled study. Medicine (Baltimore) 2016;95:e5562. 27902622 10.1097/MD.0000000000005562 PMC5134766

[14] Zhou J Yang L Yu J Efficacy of acupuncture on menstrual frequency in women with polycystic ovary syndrome: protocol for a randomized, controlled trial. Medicine (Baltimore) 2017;96:e8828. 29381988 10.1097/MD.0000000000008828 PMC5708987

[15] Leem J Kim H Jo HG Efficacy and safety of thread embedding acupuncture combined with conventional acupuncture for chronic low back pain: a study protocol for a randomized, controlled, assessor-blinded, multicenter clinical trial. Medicine (Baltimore) 2018;97:e10790. 29794761 10.1097/MD.0000000000010790 PMC6392913

[16] Hu WL Wu PC Pan LY Effect of laser acupuncture on dry eye: a study protocol for a 2-center randomized controlled trial. Medicine (Baltimore) 2018;97:e10875. 29851803 10.1097/MD.0000000000010875 PMC6393036

[17] Du J Yin J Liu L Clinical observation of 60 cases of treating cognitive disorder after cerebral injury in combination with scalp acupuncture and cognitive training. Medicine (Baltimore) 2018;97:e12420. 30290598 10.1097/MD.0000000000012420 PMC6200546

[18] Ye Y Xiao LY Liu YH Acupuncture for patients with vascular dementia: a systematic review protocol. BMJ Open 2017;7:e019066. 10.1136/bmjopen-2017-019066 PMC5728254 29217728

[19] Qin Z Liu X Yao Q Acupuncture for treating sciatica: a systematic review protocol. BMJ Open 2015;5:e007498. 10.1136/bmjopen-2014-007498 PMC4420954 25922105

[20] Zhang T Liu H Liu Z Acupuncture for neurogenic bladder due to spinal cord injury: a systematic review protocol. BMJ Open 2014;4:e006249. 10.1136/bmjopen-2014-006249 PMC4160837 25208851

[21] Wu ZS Ye XL Optic atrophy treated with acupuncture. J Tradit Chin Med 1989;9:249–50. 2630810

[22] Wei Q Gao J Treatment of optic atrophy with acupuncture. J Tradit Chin Med 1992;12:142–6. 1495340

[23] Tan Q Wang LY Wang JP Observation on improving action of acupuncture combined with Chinese medicine on visual function of optic atrophy. Zhongguo Zhen Jiu 2006;26:781–3. 17165499

[24] Liu Y Yang G Long YS Observation on therapeutic effect of acupuncture for treatment of optic atrophy. Zhongguo Zhen Jiu 2009;29:714–6. 19803238

[25] Xu L Forty-one cases of secondary optic atrophy after anti-glaucoma surgery treated with combined therapy of acupuncture and medication. Zhongguo Zhen Jiu 2012;32:689–90. 23072082

[26] Dai Y Liu M Zhang Y Meta analysis of acupuncture in the treatment of optic atrophy. Zhong Nan Da Xue Xue Bao Yi Xue Ban 2013;38:283–90. 23545824 10.3969/j.issn.1672-7347.2013.03.012

[27] Guyatt GH Oxman AD Vist GE GRADE: an emerging consensus on rating quality of evidence and strength of recommendations. BMJ 2008;336:924–6. 18436948 10.1136/bmj.39489.470347.AD PMC2335261

